# Development of an anti-pollution coating process technology for the application of an on-site PV module

**DOI:** 10.3762/bjnano.10.32

**Published:** 2019-02-01

**Authors:** Sejin Jung, Wonseok Choi, Jung Hyun Kim, Jang Myoun Ko

**Affiliations:** 1 Department of Electrical Engineering, Hanbat National University, Dajeon 34158, Republic of Korea; 2 Department of Advanced Materials Science and Engineering, Hanbat National University, Daejeon 34158, Republic of Korea; 3 Department of Chemical Engineering and Biotechnology, Hanbat National University, Daejeon 34158, Republic of Korea

**Keywords:** annealing, anti-pollution, functional film, gas torch, PV module

## Abstract

This study aimed to apply annealing processes during the coating of photovoltaic (PV) module glasses to PV modules already installed through an easy and simple procedure. Three types of annealing treatments were applied to PV module glasses, i.e., furnace, rapid thermal annealing (RTA) and torch. Among these, torch annealing, which can be easily carried out at PV module installation sites, was applied to PV module glasses using different numbers of repetition. Light transmittance, contact angle, anti-pollution characteristics, adhesion and hardness of the functional coating films after using different annealing treatment times and methods were measured, and it was confirmed that these characteristics varied depending on the annealing treatment times and methods. Through this, it was possible to optimize the process conditions that provide excellent anti-pollution characteristics and could be easily utilized at on-site PV modules.

## Introduction

The worldwide consumption of fossil fuels has caused global warming through emitting carbon dioxide (CO_2_) and greenhouse gases. To address this problem, continuous efforts have been made to reduce CO_2_ emissions through the Paris Agreement in December 2015. In addition, as fossil fuels are expected to be depleted in approximately 130 years, the development of renewable energy sources that can replace fossil energy is required. Solar energy represents the highest proportion among the renewable energy sources, and it can produce clean electricity without noise or by-products [1–2]. Photovoltaic (PV) modules are installed outdoors and are thus exposed to various external surface pollutants, such as dust, yellow dust, animal excrement and rainfall sediment. These pollutants prevent sunlight from entering the PV modules and thereby degrade the power generation efficiency. Therefore, various studies have been conducted of late to effectively prevent the surface pollution of PV modules [3–4]. The PV module surface coating materials that are currently under research must have anti-pollution functions and must endure severe temperature differences, and harsh physical (e.g., external shock) and chemical environments (e.g., animal excrement). They must also have a light transmittance of 95% or higher [5–6]. Large solar power plants are currently being installed for power generation, and a huge amount of time and cost is required for maintenance, including surface cleaning. Therefore, the introduction of a technology capable of easily removing pollutants using natural water will significantly improve the economic efficiency of the maintenance of solar power generation systems [7]. Although a self-cleaning coating technique using a photocatalyst has been developed, the durability is poor due to low adhesion and hardness. Also, production is very difficult, an energy source that causes catalytic action is needed, and the supply is low. In addition, anti-fogging and anti-condensation technologies have been applied to module production, but their anti-pollution effects are not significant [8–9].

In this study a new, easily usable coating technology is proposed that could be applied to PV modules already installed in the field to effectively improve the self-cleaning of the surfaces of PV modules. This technology should have the advantage of being applicable directly at the PV module installation site, without having to bring the module to the factory for modification. Before applying the coating technology to PV modules, glass substrates for PV modules were coated with a hydrophilic silica-based eco-friendly nanomaterial, and the coating films were thermally annealed using either a furnace, RTA or a torch. The annealing treatment that uses a torch was applied using different periods of time. For the fabricated specimens, the contact angle, anti-pollution characteristics, hardness, and adhesion were measured. The process conditions were optimized by analyzing the measurement results.

## Experimental

The coating solution that was used to improve the anti-pollution characteristics of the PV modules contained silicon dioxide (SiO_2_), lithium (Li), and potassium (K). The viscosity, density, and specific gravity (referring to the density of water) of the coating solution were 0.01–0.03 kg/m·s, 1.1 g/cm^3^, and 1.13 ± 0.05, respectively. The solution can be used to coat various materials, such as metals, ceramics, and glass [9].

Before coating the glass slide substrates, the substrates were subjected to ultrasonic cleaning for 10 min in, consecutively, trichloroethylene, acetone, methanol and deionized water (DI water). The substrates were coated with the coating solution using a brush. After being dried at room temperature for 20 min, the substrates were thermally annealed using a furnace (L-Series, Jeio Tech Co., South Korea), RTA (RTP-1200, Nextron Co., South Korea), and a gas torch (KT-2211, Kovea Co., South Korea, using butane gas). The length of the flame was about 10 cm, the distance between the flame and the specimen was about 5 cm, and the temperature was about 300 °C. In addition, the annealing treatment that uses a gas torch, which can be easily utilized to surfaces of installed or operating PV modules, was applied from one to five times.

The contact angles of the fabricated functional coating films were measured using a contact angle analyzer (Phoenix 300 Touch, S.E.O. Co., South Korea). The anti-pollution characteristics were measured using permanent markers instead of actual pollutants. This method is useful for checking the level of pollutant removal. The hardness was measured using a hardness tester (CT-PC1, CORETECH Co., South Korea) equipped with pencils with hardness values from H to 9H (Mitsubishipencil Co., Ltd., Japan) in accordance with ASTM D3363 of the American Society for Testing and Materials (ASTM). The optical characteristics were measured using the integrating sphere of a UV–visible spectrophotometer (Mega 700, Scinco Co., Ltd., South Korea).

## Results and Discussion

**Figure 1:**
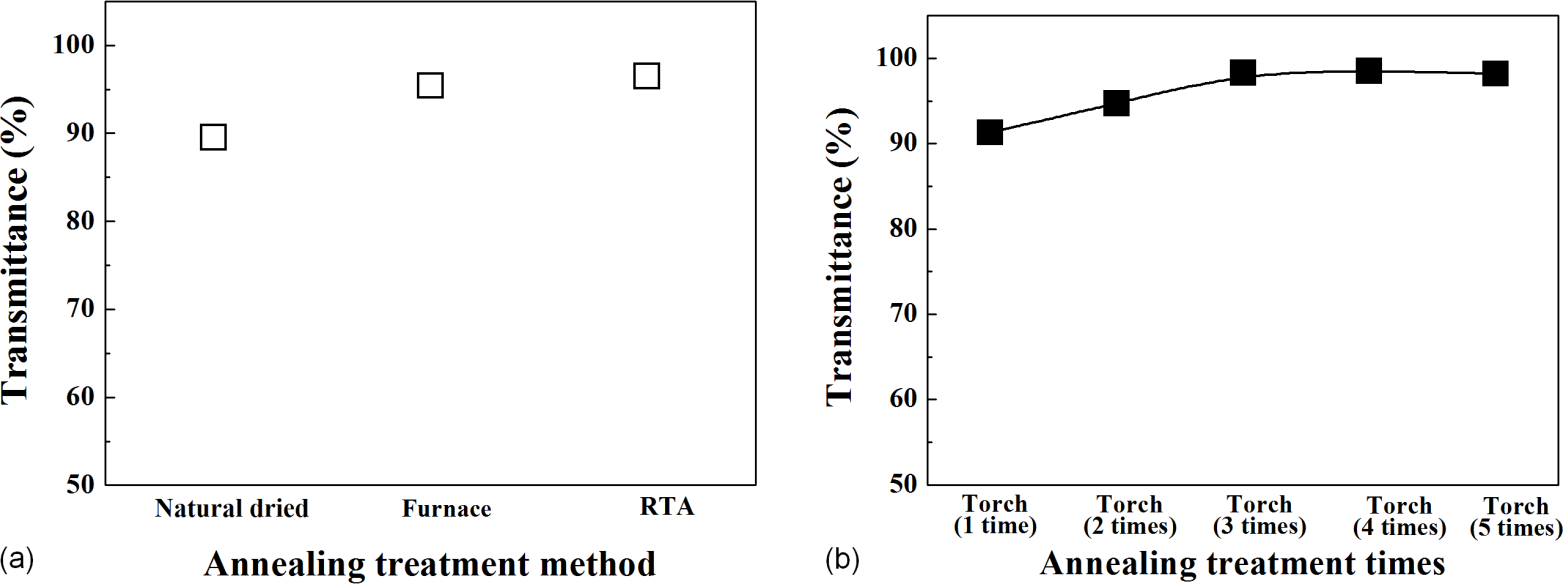
Transmittance (a) according to the annealing treatment method and (b) according to the number of annealing treatment using a gas torch.

The light transmittance measurement results of the fabricated functional coatings are summarized in Figure 1. The light transmittance of the coating film thermally annealed at 300 °C using a furnace was determined to be 95.4%, and that of the coating film thermally annealed at 300 °C using RTA, 96.5%. When annealing treatment was performed one to five times using a torch, the light transmittance was found to be 91.3, 94.7, 98.3, 98.5, and 98.2%, respectively. The light transmittance increased for the first three times of torch annealing. After the fourth and fifth treatment the transmittance was similar to that after the third annealing.

**Figure 2:**
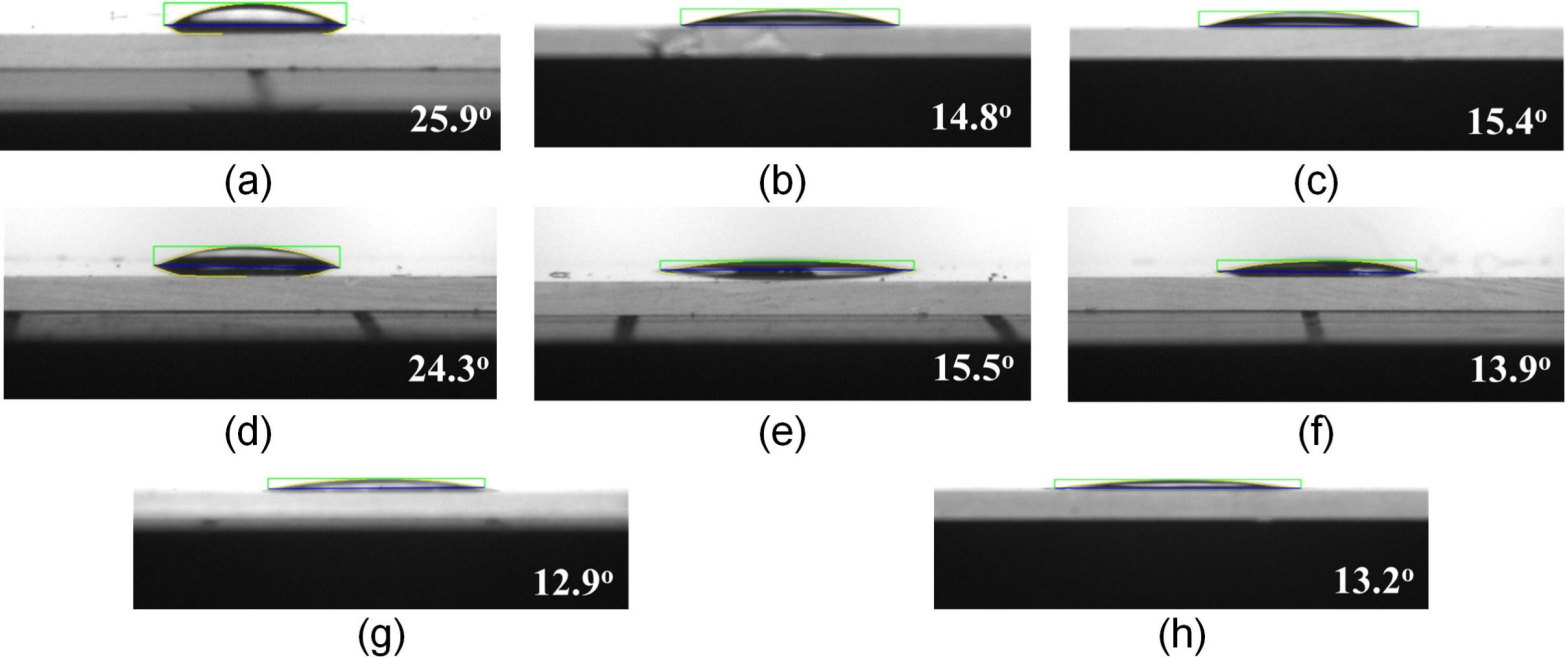
Contact angles of the coating films fabricated using various annealing treatment methods: (a) naturally dried; (b) annealed with RTA; (c) annealed with a furnace; (d) annealed once with a gas torch; (e) annealed two times with a gas torch; (f) annealed three times with a gas torch; (g) annealed four times a with gas torch; and (h) annealed five times with a gas torch.

**Figure 3:**
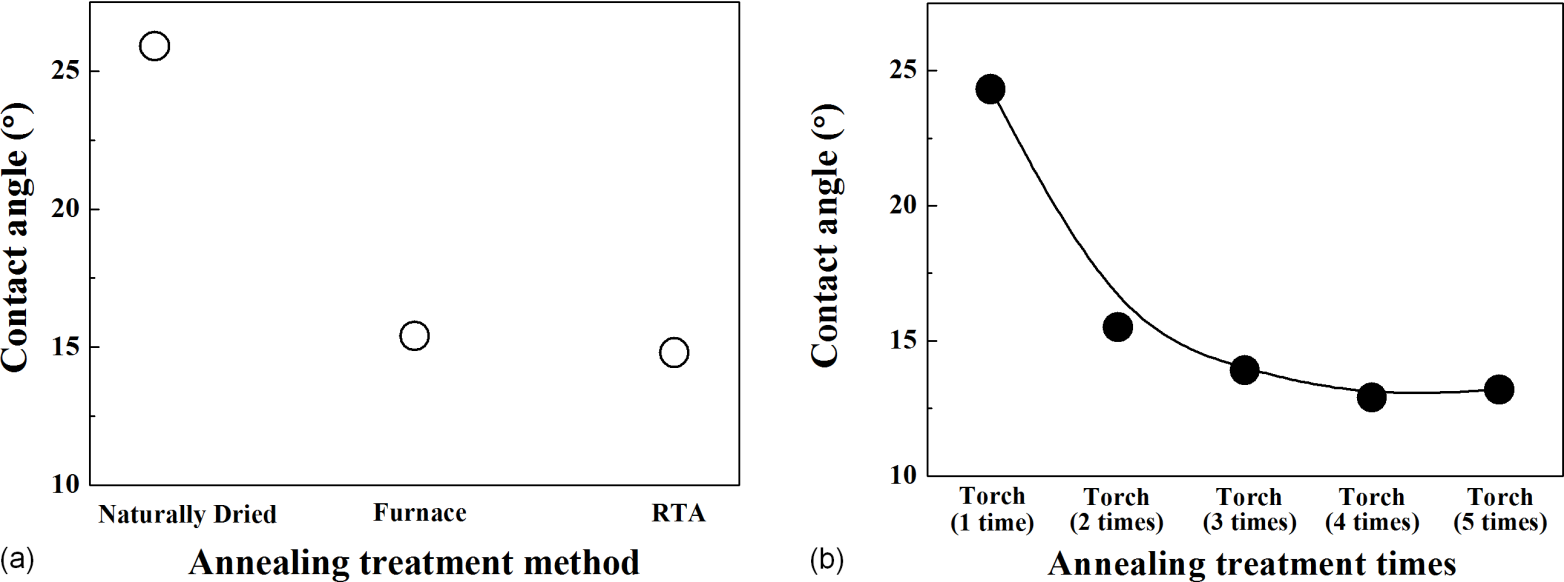
Contact angle by (a) annealing treatment method and (b) annealing treatment times using a gas torch.

Figure 2 and Figure 3 show the contact angle characteristics according to the annealing treatment method. The contact angle was 15.4° when the coating film was thermally annealed using a furnace, and it was 14.8° when the coating film was thermally annealed using RTA. When annealing treatment was performed one to three times using a torch, the contact angle was 24.3°, 15.5°, and 13.9°, respectively, but the contact angles in the fourth and fifth annealing treatments were similar to that in the third annealing treatment.

**Figure 4:**
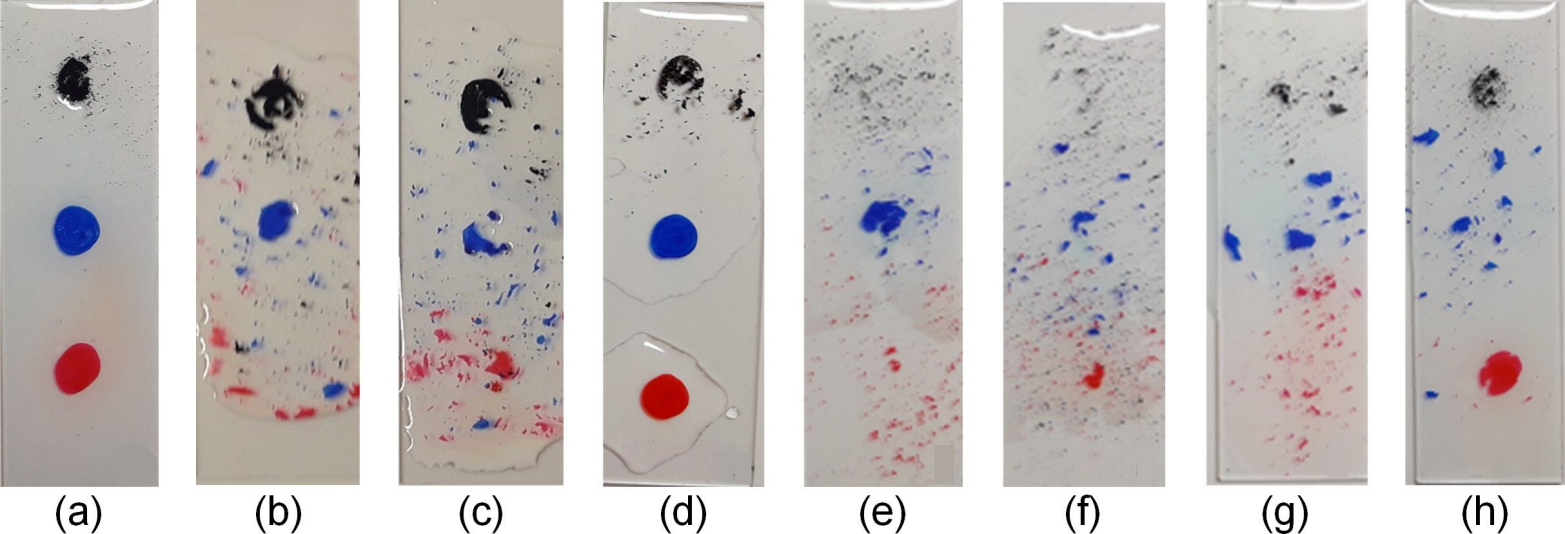
Anti-pollution characteristics of the coating films fabricated using various annealing treatment methods: (a) naturally dried; (b) annealed with RTA; (c) annealed with a furnace; (d) annealed once with a gas torch; (e) annealed two times with a gas torch; (f) annealed three times with a gas torch; (g) annealed four times with a gas torch; and (h) annealed five times with a gas torch.

Figure 4 shows the anti-pollution characteristics after annealing treatment. Black, red, and blue markings were applied on the glass slide substrates coated with the functional coating solution, using oil pens. After the markings were naturally dried and cleaned with water, the specimens thermally annealed using a furnace, RTA, and three to five torch applications showed excellent anti-pollution characteristics. In particular, the specimens thermally annealed using three to five torch applications exhibited the most excellent characteristics.

**Figure 5:**
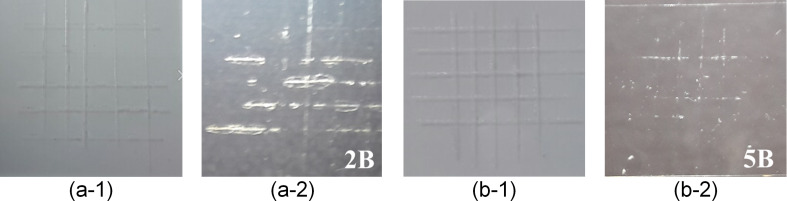
Adhesion characteristics of the coating films fabricated using various annealing treatment methods: (a-1) naturally dried; (a-2) coating film after the adhesion test; (b-1) annealed; and (b-2) coating film after the adhesion test.

**Figure 6:**
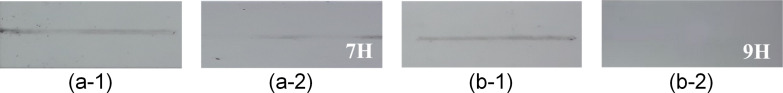
Hardness characteristics of the coating films fabricated using various annealing treatment methods: (a-1) naturally dried; (a-2) coating film after the hardness test; (b-1) annealed; and (b-2) coating film after the hardness test.

Figure 5 and Figure 6 show the hardness and adhesion characteristics when annealing treatment was performed on the coating films using a furnace, RTA, and one to five torch applications. For the analysis of the characteristics, the hardness values of the fabricated films were measured using a pencil hardness tester, in accordance with the ASTM D3363 criteria, and the adhesion values were measured in accordance with the ASTM D3359 criteria. All the annealed coating films exhibited 5B adhesion and 9H hardness, while the coating film that had been dried at room temperature exhibited 2B adhesion and 7H hardness.

## Conclusion

A functional coating was applied to glass slide substrates to improve their anti-pollution characteristics for application in PV modules. After coating glass slide substrates made of the same material as glass substrates for PV modules with functional films, the results of naturally drying them or thermally annealing them with a furnace, RTA, or a torch were compared. In addition, annealing treatments with a torch were carried out one to five times to observe the changes in the sample characteristics. For the analysis of the characteristics of the fabricated specimens, light transmittance, contact angle, anti-pollution characteristics, hardness, and adhesion were measured.

The thermally annealed specimens showed 91.3% or higher light transmittance. In particular, when annealing using a torch three to four times, high light transmittance values of 98.3 and 98.5% were obtained. For the results of the contact angle analysis, the thermally annealed specimens showed a contact angle of 24.3° or lower. In particular, when annealing using a torch four to five times, the contact angles were 12.9 and 13.2°, respectively, indicating improvements in the hydrophilic characteristics. The analysis of the anti-pollution characteristics revealed that all the thermally annealed specimens exhibited improved anti-pollution characteristics. In particular, it was confirmed that the anti-pollution characteristics improved as the contact angle decreased. As for the measurement results of the hardness and adhesion, which are mechanical characteristics, all the thermally annealed substrates exhibited 9H hardness and 5B adhesion results, but the coating film that had been dried at room temperature showed 7H and 2B values. These results indicate that annealing treatment with a torch produces results similar to or better than those produced by annealing treatment with a furnace or RTA. Therefore, if the surfaces of PV modules installed outdoors are coated and thermally annealed with a torch, the annealing treatment process will be faster and easier. The results of this study confirm that the annealing treatment process using a torch can be applied directly to the installed PV module. On-site PV modules can be immediately processed without having to go to the factory, so it is expected that the amount of maintenance work will be reduced and that the economic efficiency will increase.

## Supporting Information

File 1 Overview.
