# Supplementary material for: Development of an anti-pollution coating process technology for the application of an on-site PV module
Source: Beilstein J Nanotechnol. 2019 Feb 1;10:332–6. doi: 10.3762/bjnano.10.32 (PMC6369985; doi:10.3762/bjnano.10.32)
Supplement: File 1 — Overview. [file Beilstein_J_Nanotechnol-10-332-s001.pdf]

BEILSTEIN JOURNAL OF NANOTECHNOLOGY

Supporting Information

for

Development of an anti-pollution coating process technology for the application of an on-site PV module

Sejin Jung, Wonseok Choi, Jung Hyun Kim and Jang Myoun Ko

Beilstein J. Nanotechnol. **2019,** *10,* 332–336. doi:10.3762/bjnano.10.32

Overview

License and Terms: This is a supporting information file under the terms of the Creative Commons Attribution License (http://creativecommons.org/licenses/by/4.0). Please note that the reuse, redistribution and reproduction in particular requires that the authors and source are credited.
The license is subject to the *Beilstein Journal of Nanotechnology* terms and conditions: (https://www.beilstein-journals.org/bjnano)

Development of Anti-pollution Coating Process Technology for the Application of Onsite PV Module

Experiment

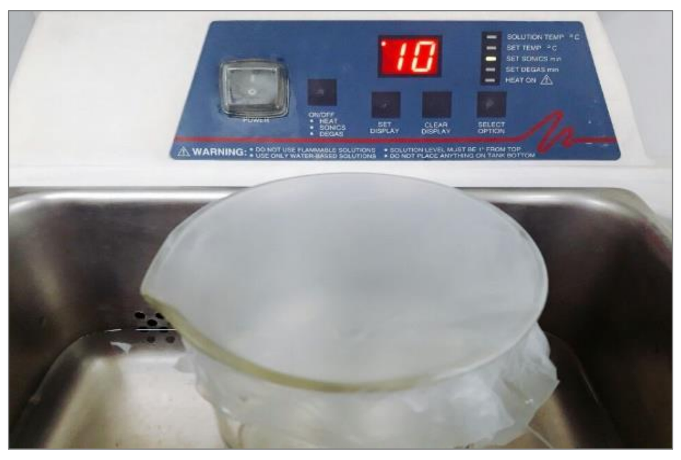
Cleaning Glass

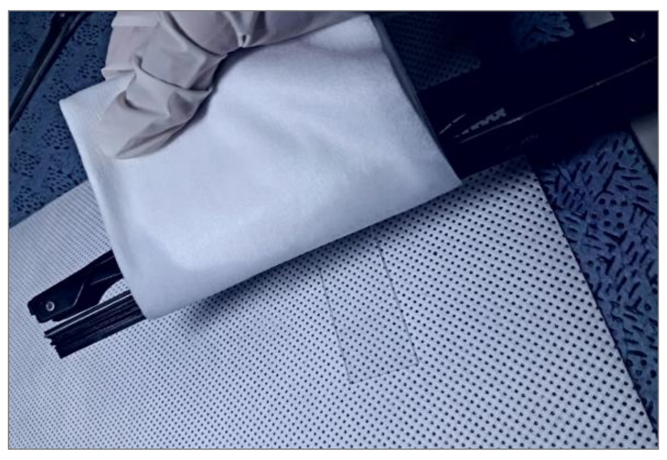
Brush Coating on Glass

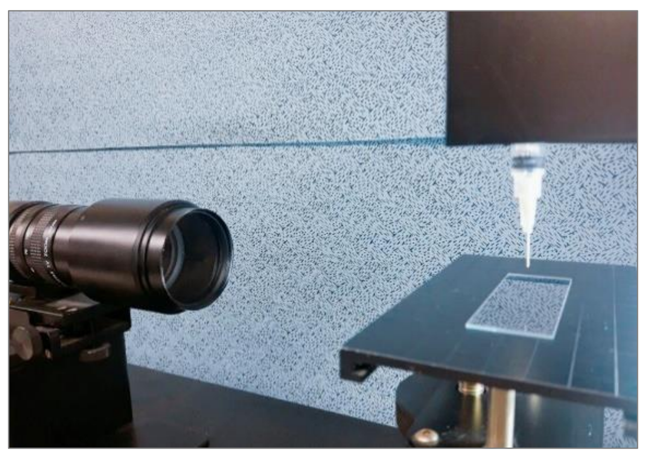
Properties analysis

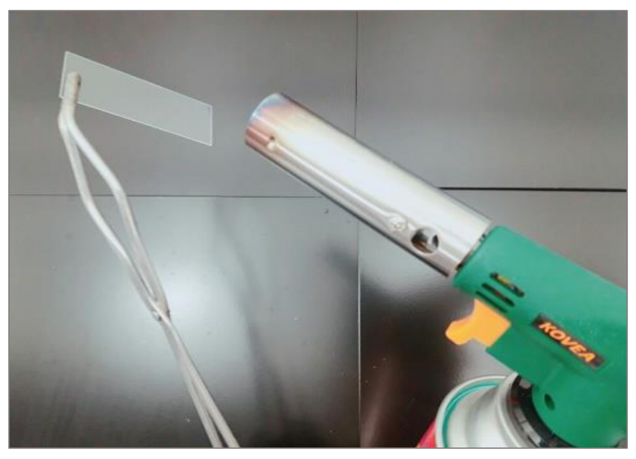
Annealing

Condition	Number of annealing treatment											
	once		twice				third times					
	Heating time	Cooling time	Heating time	Cooling time	Heating time	Cooling time	Heating time	Cooling time	Heating time	Cooling time	Heating time	Cooling time
Anneal 1	2min	10min	2min	10min	2min	10min	2min	10min	2min	10min	2min	10min
Anneal 2	2min	10min	2min	10min	2min	10min	2min	10min	2min	10min	2min	10min
Anneal 3	2min	10min	2min	10min	2min	10min	2min	10min	2min	10min	2min	10min

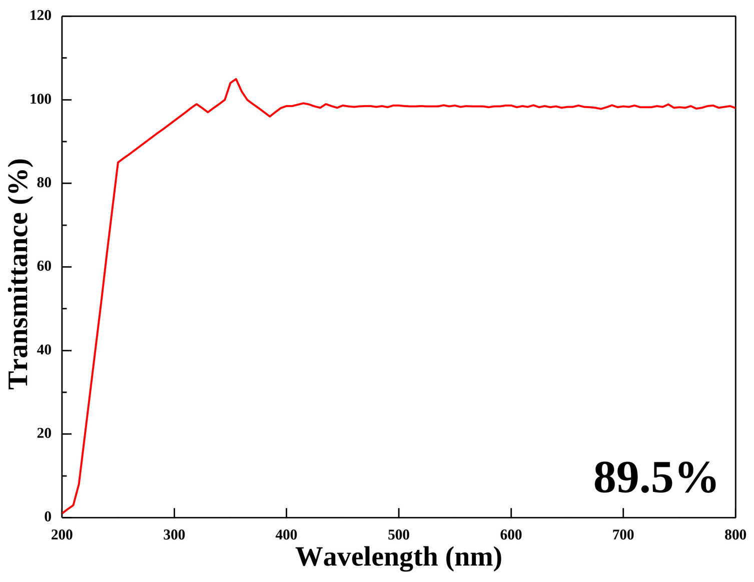

Room temperature

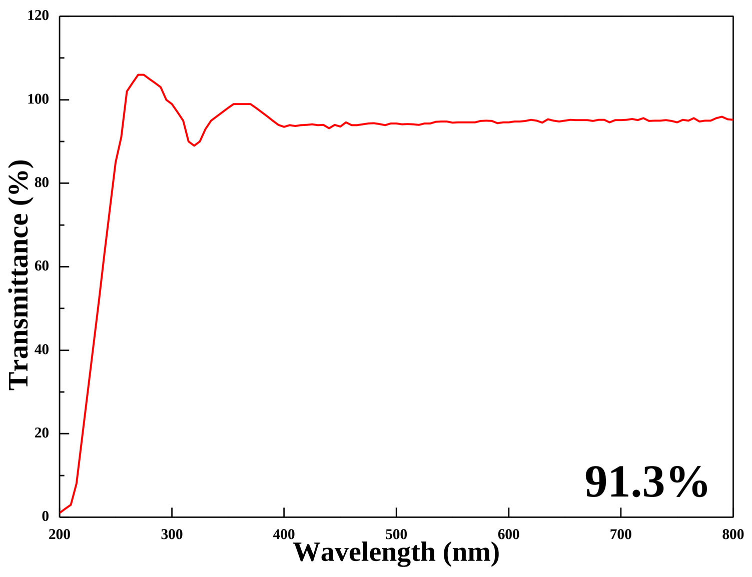

Anneal 1

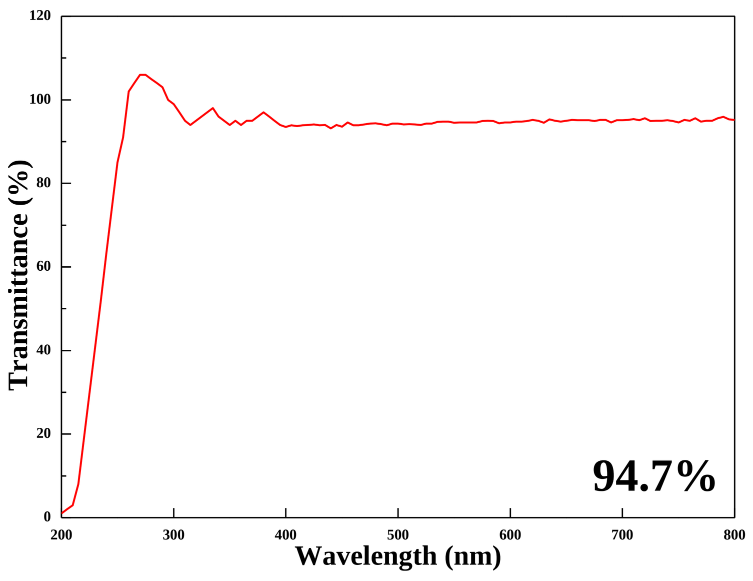

Anneal 2

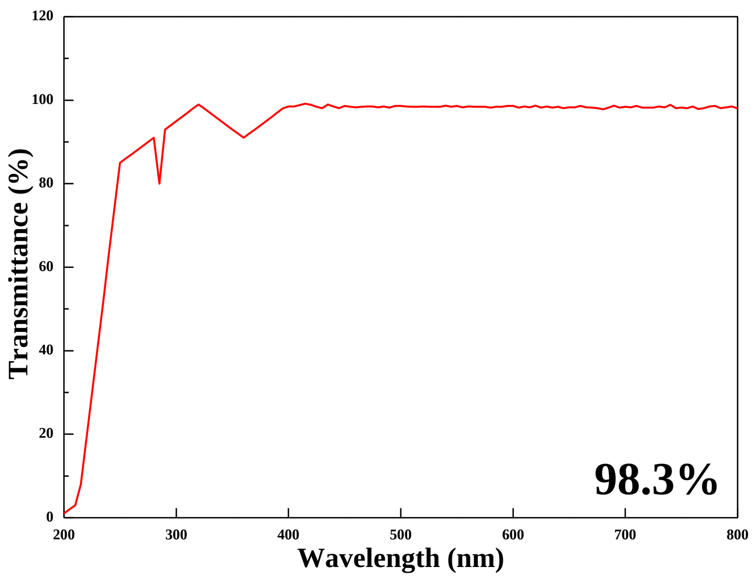

Anneal 3

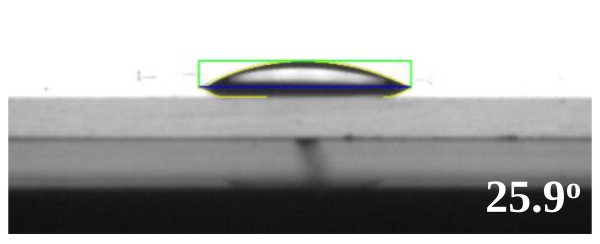

Room temperature

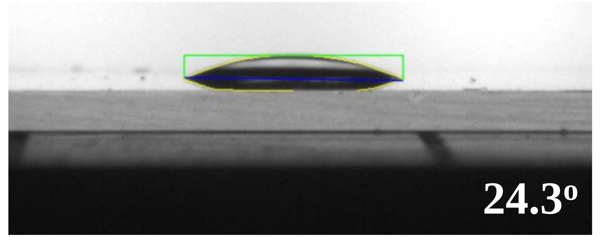

Anneal 1

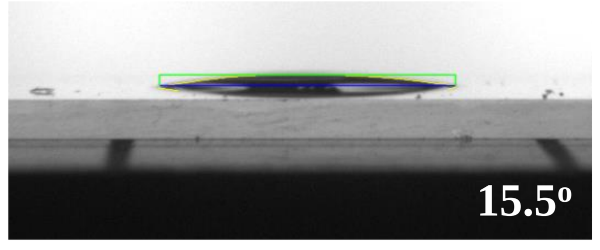

Anneal 2

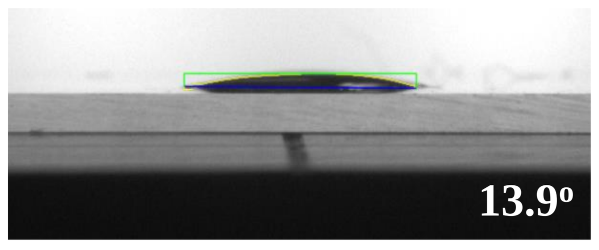

Anneal 3

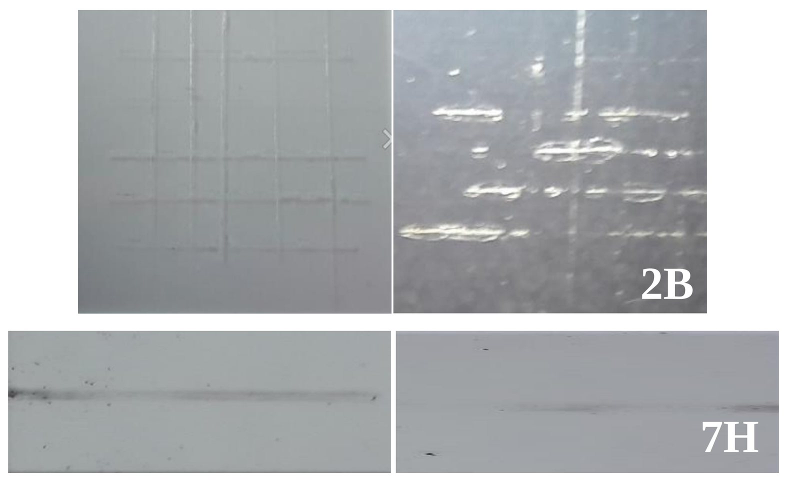

Room temperature

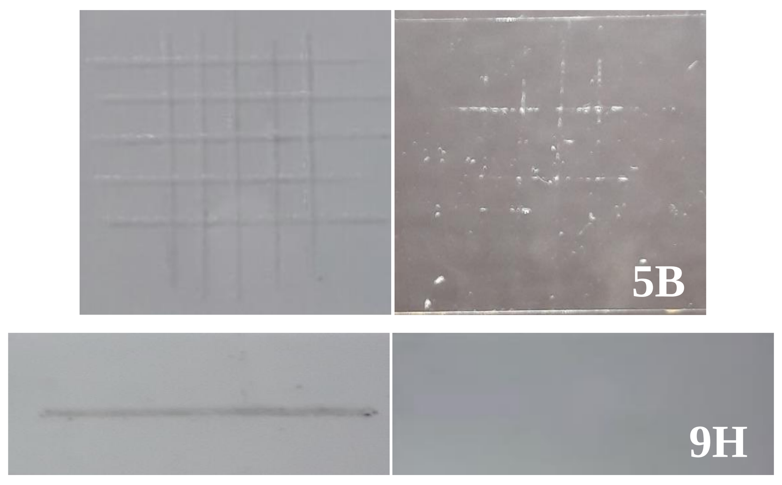

Anneal 1, Anneal 2 and Anneal 3

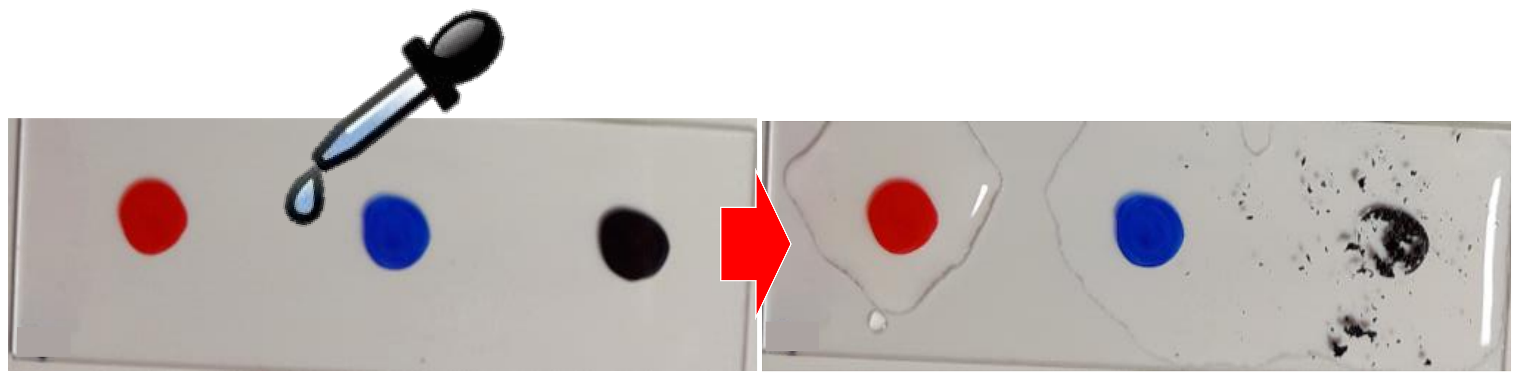
Dry at the room temperature (No annealing treatment)

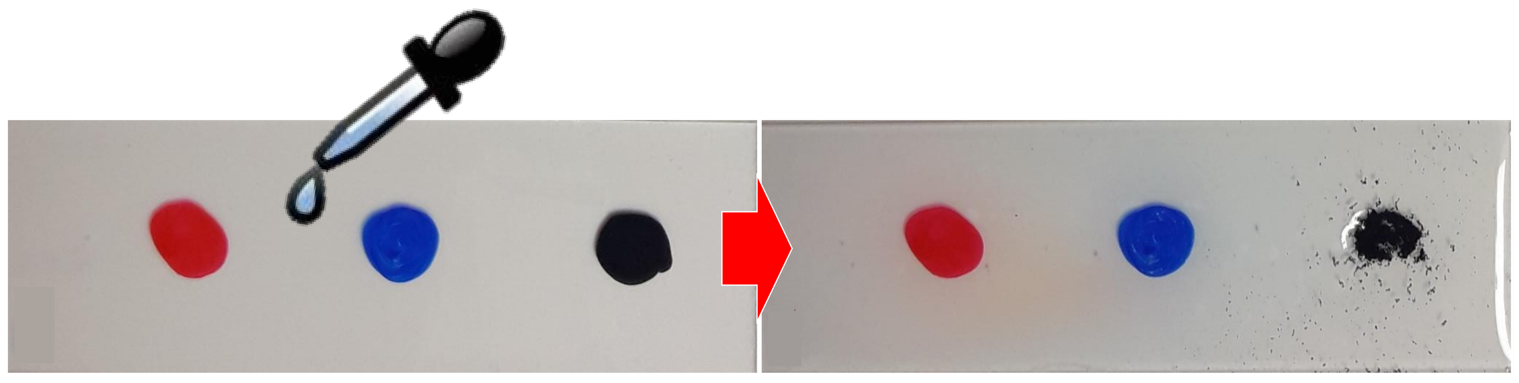
Anneal 1 (Annealing treatment proceeded once)

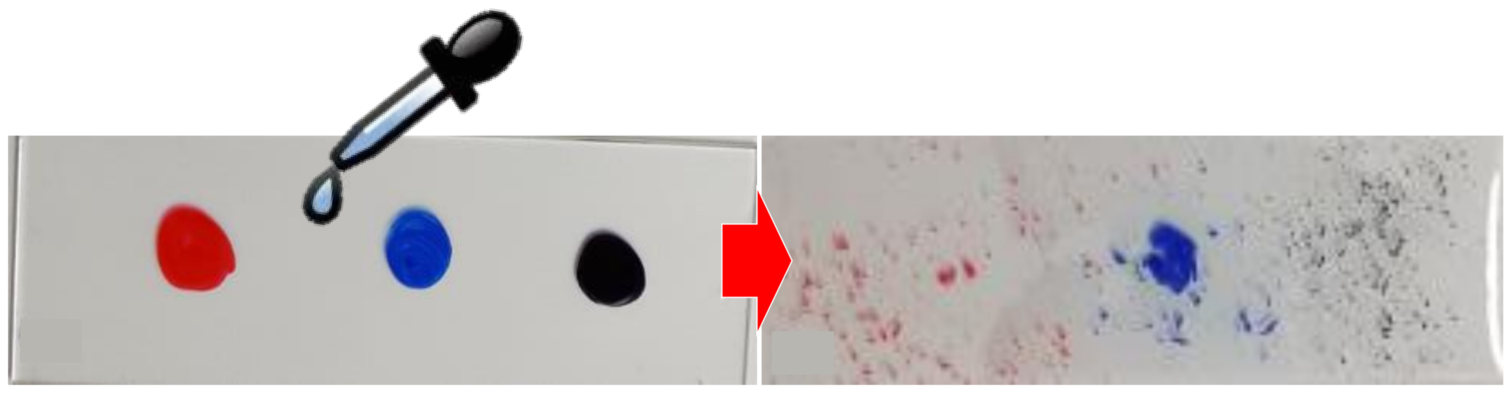
Anneal 2 (Annealing treatment proceeded twice)

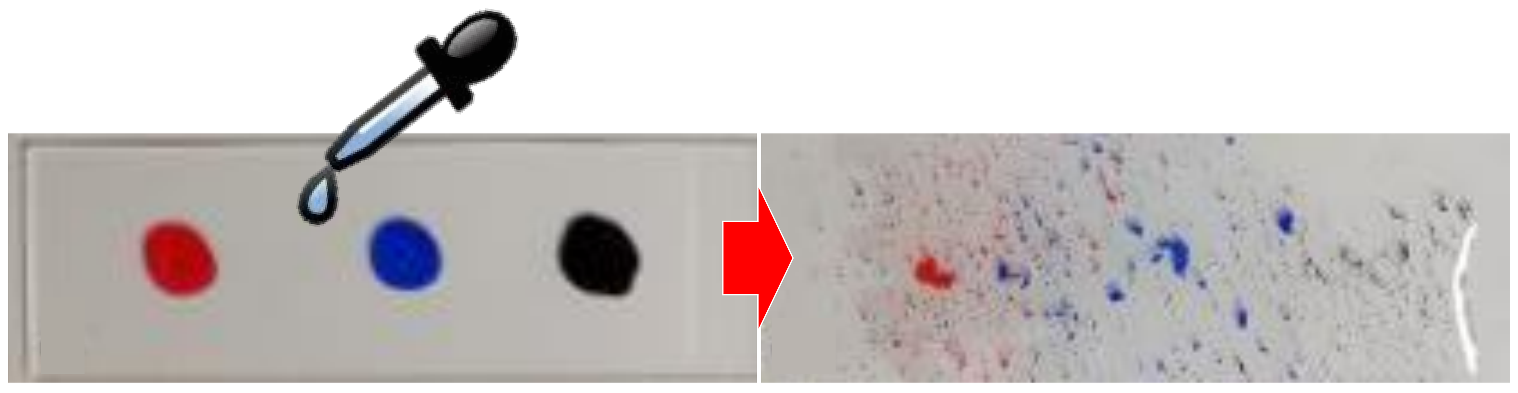
Anneal 3 (Annealing treatment proceeded third times)

Conclusions

- This study was conducted to investigate the possibility of utilizing the technology that exhibits excellent anti-pollution characteristics in the field.
- When the annealing treatment was performed more than once, the transmittance was higher than 91%, and hardness and adhesion were found to be the highest grade.
- In the condition that the annealing treatment was repeated three times, the anti-pollution characteristic was the best.
- The technology presented in this study has advantages that it can be processed in the field and has excellent usability.
- The developed technology is expected to be utilized not only in the PV module but also in the field were the anti-pollution characteristic is required.
